# 4,4,5,5-Tetramethyl-2-(4-pyridinio)imidazoline-1-oxyl-3-oxide chloride

**DOI:** 10.1107/S1600536808043158

**Published:** 2008-12-24

**Authors:** Jiu Li Chang, Zhi Yong Gao, Kai Jiang

**Affiliations:** a College of Chemistry and Environmental Science, Henan Normal University, Xinxiang, 453002, People’s Republic of China

## Abstract

The title compound C_12_H_17_N_3_O_2_^+^·Cl^−^ consists of a discrete [NITpPyH]^+^ cation [NITpPy = 2-(4′-pyrid­yl)-4,4,5,5-tetra­methyl­imidazoline-1-oxyl-3-oxide] and a chloride anion. The NITpPy mol­ecule is protonated at the N atom of the pyridyl ring. The anions and cations are connected *via* N—H⋯Cl hydrogen bonds.

## Related literature

For the design and synthesis of mol­ecule-based magnetic materials, see: Bogani *et al.* (2005 ▶); Wang *et al.* (2004 ▶). For nitronyl nitroxide radicals (NITR), see: Fettouhi *et al.* (2003 ▶). For related literature, see: Stroh *et al.* (1999 ▶); Hirel *et al.* (2001 ▶); Chang *et al.* (2005 ▶); Wang *et al.* (2003 ▶). For the synthesis of the title compound see: Ullman *et al.* (1970 ▶, 1972 ▶)
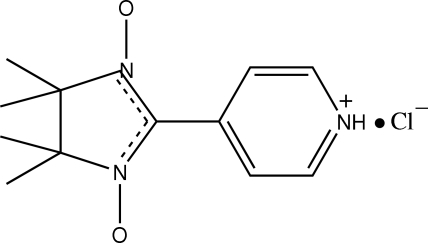


## Experimental

### 

#### Crystal data

C_12_H_17_N_3_O_2_^+^·Cl^−^
*M* *_r_* = 270.74
Monoclinic, 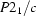

*a* = 10.863 (14) Å
*b* = 11.927 (15) Å
*c* = 11.130 (15) Å
β = 102.81 (2)°
*V* = 1406 (3) Å^3^
*Z* = 4
Mo *K*α radiation
μ = 0.27 mm^−1^
*T* = 291 (2) K
0.30 × 0.26 × 0.23 mm

#### Data collection

Bruker SMART CCD area-detector diffractometer
Absorption correction: multi-scan (*SADABS*; Sheldrick, 1996 ▶) *T*_min_ = 0.923, *T*_max_ = 0.939
7172 measured reflections
2609 independent reflections
2120 reflections with *I* > 2σ(*I*)
*R*_int_ = 0.037

#### Refinement

*R*[*F* ^2^ > 2σ(*F* ^2^)] = 0.048
*wR*(*F* ^2^) = 0.140
*S* = 1.03
2609 reflections
167 parameters
H-atom parameters constrained
Δρ_max_ = 0.44 e Å^−3^
Δρ_min_ = −0.22 e Å^−3^

### 

Data collection: *SMART* (Bruker, 2002 ▶); cell refinement: *SAINT* (Bruker, 2002 ▶); data reduction: *SAINT*; program(s) used to solve structure: *SHELXS97* (Sheldrick, 2008 ▶); program(s) used to refine structure: *SHELXL97* (Sheldrick, 2008 ▶); molecular graphics: *SHELXTL* (Sheldrick, 2008 ▶); software used to prepare material for publication: *publCIF* (Westrip, 2009 ▶).

## Supplementary Material

Crystal structure: contains datablocks I, New_Global_Publ_Block. DOI: 10.1107/S1600536808043158/bx2189sup1.cif

Structure factors: contains datablocks I. DOI: 10.1107/S1600536808043158/bx2189Isup2.hkl

Additional supplementary materials:  crystallographic information; 3D view; checkCIF report

## Figures and Tables

**Table 1:** Hydrogen-bond geometry (Å, °)

*D*—H⋯*A*	*D*—H	H⋯*A*	*D*⋯*A*	*D*—H⋯*A*
N1—H1*D*⋯Cl1^i^	0.86	2.17	3.028 (3)	174

Symmetry code: (i) 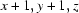.

**Fig. 1.**
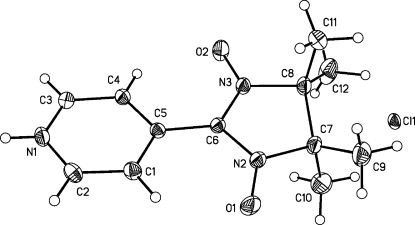
ORTEP drawing of the title compound with atom labeling. The thermal ellipsoids are drawn at 30% probability level.

## supplementary crystallographic information

### Comment

The design and synthesis of molecule-based magnetic materials is one of the major subjects of materials science in which the combination of metal ions and organic radicals are used to construct assembled systems (Bogani *et al.*, 2005; Wang *et al.*, 2004). Nitronyl nitroxide radicals (NITR), independently or in combination with metal ions, have been one of the most widely studied systems in molecular magnetism for understanding the radical-radical or metal-radical as well as for synthesizing organic ferromagnets and metal-radical magnetic materials (Fettouhi *et al.*, 2003). However, to our knowledge so far few charge transfer complexes of nitronyl nitroxide radicals used as proton receptor have been reported. In order to better understand the behavior of proton transfer in charge transfer complexes, the synthesis and crystal structure of the title compound have been investigated. The structure of the title compound is shown in Fig. 1. The NITpPy molecule is protonated at N atom of the pyridyl ring by accepting a proton from the acid solution. The transfer of protons result in a intermolecular hydrogen bond between NITpPy and chloride.The anions and cations are connected *via* N—H···Cl hydrogen bonds. The nitronyl nitroxide fragment O—N—C—N—O is almost coplanar, but make a dihedral angle of 8.6 (2)° with the pyridyl ring.

### Experimental

NITpPy was synthesized according to a literature procedure (Ullman *et al.*,1970; Ullman *et al.*, 1972). Single crystals of the title compound suitable for X-ray measurements were obtained by recrystallization from acetonitrile solution and HCl 10:1 (*v*/*v*) solution at room temperature.

### Refinement

The H atoms were positioned geometrically and refined using the riding-model approximation, with C—H = 0.93 or 0.96 Å and N—H = 0.96 Å and *U*_iso_(H) = 1.2U_eq_(carrier) or *U*_iso_(H) = 1.5U_eq_(methyl carrier).

### Crystal data

**Table:**

C_12_H_17_N_3_O_2_^+^·Cl^−^	*F*(000) = 572
*M**_r_* = 270.74	*D*_x_ = 1.279 Mg m^−^^3^
Monoclinic, *P*2_1_/*c*	Mo *K*α radiation, λ = 0.71073 Å
Hall symbol: -P 2ybc	Cell parameters from 3005 reflections
*a* = 10.863 (14) Å	θ = 2.5–27.3°
*b* = 11.927 (15) Å	µ = 0.27 mm^−^^1^
*c* = 11.130 (15) Å	*T* = 291 K
β = 102.81 (2)°	BLOCK, black
*V* = 1406 (3) Å^3^	0.30 × 0.26 × 0.23 mm
*Z* = 4	

### Data collection

**Table:**

Bruker SMART CCD area-detector diffractometer	2609 independent reflections
Radiation source: fine-focus sealed tube	2120 reflections with *I* > 2σ(*I*)
graphite	*R*_int_ = 0.037
phi and ω scans	θ_max_ = 25.5°, θ_min_ = 2.5°
Absorption correction: multi-scan (*SADABS*; Sheldrick, 1996)	*h* = −13→11
*T*_min_ = 0.923, *T*_max_ = 0.939	*k* = −13→14
7172 measured reflections	*l* = −13→13

### Refinement

**Table:**

Refinement on *F*^2^	Primary atom site location: structure-invariant direct methods
Least-squares matrix: full	Secondary atom site location: difference Fourier map
*R*[*F*^2^ > 2σ(*F*^2^)] = 0.048	Hydrogen site location: inferred from neighbouring sites
*wR*(*F*^2^) = 0.140	H-atom parameters constrained
*S* = 1.03	*w* = 1/[σ^2^(*F*_o_^2^) + (0.0696*P*)^2^ + 0.6783*P*] where *P* = (*F*_o_^2^ + 2*F*_c_^2^)/3
2609 reflections	(Δ/σ)_max_ = 0.001
167 parameters	Δρ_max_ = 0.44 e Å^−^^3^
0 restraints	Δρ_min_ = −0.22 e Å^−^^3^

### Special details

**Table:**

Geometry. All e.s.d.'s (except the e.s.d. in the dihedral angle between two l.s. planes)are estimated using the full covariance matrix. The cell e.s.d.'s are takeninto account individually in the estimation of e.s.d.'s in distances, anglesand torsion angles; correlations between e.s.d.'s in cell parameters are onlyused when they are defined by crystal symmetry. An approximate (isotropic)treatment of cell e.s.d.'s is used for estimating e.s.d.'s involving l.s. planes.
Refinement. Refinement of *F*^2^ against ALL reflections. The weighted *R*-factor *wR* andgoodness of fit *S* are based on *F*^2^, conventional *R*-factors *R* are basedon *F*, with *F* set to zero for negative *F*^2^. The threshold expression of*F*^2^ > σ(*F*^2^) is used only for calculating *R*-factors(gt) *etc*. and isnot relevant to the choice of reflections for refinement. *R*-factors basedon *F*^2^ are statistically about twice as large as those based on *F*, and *R*-factors based on ALL data will be even larger.

### Fractional atomic coordinates and isotropic or equivalent isotropic displacement parameters (Å^2^)

**Table:**

	*x*	*y*	*z*	*U*_iso_*/*U*_eq_	
Cl1	0.05896 (6)	0.43910 (4)	0.31130 (6)	0.0548 (2)	
O1	0.64998 (19)	1.16127 (15)	0.74894 (19)	0.0700 (6)	
O2	0.78794 (19)	0.86362 (13)	0.53807 (17)	0.0633 (5)	
N1	0.92752 (17)	1.26424 (15)	0.43535 (17)	0.0452 (5)	
H1D	0.9687	1.3101	0.3992	0.054*	
N2	0.67191 (18)	1.06169 (14)	0.71219 (17)	0.0425 (4)	
N3	0.73993 (17)	0.92068 (14)	0.61433 (16)	0.0395 (4)	
C1	0.8082 (2)	1.23103 (17)	0.5864 (2)	0.0437 (5)	
H1	0.7700	1.2586	0.6473	0.052*	
C2	0.8735 (2)	1.30278 (18)	0.5247 (2)	0.0479 (6)	
H2	0.8799	1.3784	0.5454	0.057*	
C3	0.9190 (2)	1.15584 (18)	0.4007 (2)	0.0437 (5)	
H3	0.9562	1.1321	0.3375	0.052*	
C4	0.8550 (2)	1.07904 (17)	0.45867 (19)	0.0391 (5)	
H4	0.8484	1.0045	0.4336	0.047*	
C5	0.80009 (18)	1.11518 (16)	0.55597 (18)	0.0343 (4)	
C6	0.73824 (19)	1.03513 (16)	0.62469 (18)	0.0348 (5)	
C7	0.6107 (2)	0.95916 (19)	0.7557 (2)	0.0446 (5)	
C8	0.6900 (2)	0.86274 (18)	0.7158 (2)	0.0479 (6)	
C9	0.6147 (3)	0.9682 (3)	0.8936 (3)	0.0755 (9)	
H9A	0.7009	0.9699	0.9387	0.113*	
H9B	0.5727	0.9047	0.9193	0.113*	
H9C	0.5729	1.0358	0.9093	0.113*	
C10	0.4731 (3)	0.9600 (3)	0.6823 (3)	0.0760 (9)	
H10A	0.4336	1.0290	0.6972	0.114*	
H10B	0.4285	0.8981	0.7078	0.114*	
H10C	0.4713	0.9533	0.5959	0.114*	
C11	0.8094 (3)	0.8328 (3)	0.8176 (3)	0.0673 (8)	
H11A	0.8630	0.7836	0.7837	0.101*	
H11B	0.7839	0.7962	0.8849	0.101*	
H11C	0.8546	0.9002	0.8465	0.101*	
C12	0.6206 (4)	0.7561 (2)	0.6664 (3)	0.0855 (11)	
H12A	0.5572	0.7731	0.5938	0.128*	
H12B	0.5814	0.7245	0.7279	0.128*	
H12C	0.6795	0.7031	0.6462	0.128*	

### Atomic displacement parameters (Å^2^)

**Table:**

	*U*^11^	*U*^22^	*U*^33^	*U*^12^	*U*^13^	*U*^23^
Cl1	0.0756 (5)	0.0354 (3)	0.0595 (4)	−0.0077 (2)	0.0278 (3)	0.0030 (2)
O1	0.0900 (14)	0.0420 (9)	0.0963 (15)	0.0035 (9)	0.0597 (12)	−0.0127 (9)
O2	0.0960 (14)	0.0354 (8)	0.0746 (12)	−0.0055 (8)	0.0536 (11)	−0.0104 (8)
N1	0.0455 (11)	0.0403 (10)	0.0500 (11)	−0.0080 (8)	0.0114 (9)	0.0098 (8)
N2	0.0454 (11)	0.0370 (9)	0.0502 (11)	0.0003 (7)	0.0215 (9)	−0.0023 (7)
N3	0.0471 (11)	0.0319 (8)	0.0438 (10)	−0.0033 (7)	0.0197 (8)	−0.0025 (7)
C1	0.0529 (14)	0.0357 (11)	0.0439 (12)	−0.0023 (9)	0.0134 (10)	−0.0043 (9)
C2	0.0575 (15)	0.0315 (10)	0.0526 (14)	−0.0059 (9)	0.0082 (11)	−0.0007 (9)
C3	0.0432 (13)	0.0431 (11)	0.0476 (12)	0.0019 (9)	0.0159 (10)	0.0056 (9)
C4	0.0428 (12)	0.0329 (10)	0.0439 (12)	−0.0007 (8)	0.0142 (10)	0.0002 (8)
C5	0.0331 (11)	0.0316 (9)	0.0378 (11)	−0.0006 (8)	0.0065 (8)	0.0010 (8)
C6	0.0356 (11)	0.0329 (10)	0.0377 (11)	−0.0004 (8)	0.0115 (9)	−0.0010 (8)
C7	0.0433 (13)	0.0459 (12)	0.0495 (13)	−0.0048 (9)	0.0206 (10)	0.0017 (9)
C8	0.0563 (14)	0.0375 (11)	0.0575 (14)	−0.0053 (10)	0.0288 (12)	0.0041 (9)
C9	0.102 (3)	0.0758 (19)	0.0605 (17)	0.0035 (17)	0.0428 (17)	0.0043 (14)
C10	0.0460 (16)	0.080 (2)	0.102 (3)	−0.0054 (14)	0.0163 (16)	0.0114 (17)
C11	0.0678 (18)	0.0642 (16)	0.0743 (18)	0.0131 (13)	0.0250 (15)	0.0262 (14)
C12	0.103 (3)	0.0554 (16)	0.116 (3)	−0.0362 (17)	0.064 (2)	−0.0199 (17)

### Geometric parameters (Å, °)

**Table:**

O1—N2	1.295 (3)	C7—C9	1.529 (4)
O2—N3	1.285 (2)	C7—C10	1.536 (4)
N1—C2	1.343 (3)	C7—C8	1.559 (3)
N1—C3	1.346 (3)	C8—C12	1.518 (4)
N1—H1D	0.8600	C8—C11	1.563 (4)
N2—C6	1.371 (3)	C9—H9A	0.9600
N2—C7	1.522 (3)	C9—H9B	0.9600
N3—C6	1.370 (3)	C9—H9C	0.9600
N3—C8	1.523 (3)	C10—H10A	0.9600
C1—C2	1.387 (3)	C10—H10B	0.9600
C1—C5	1.421 (3)	C10—H10C	0.9600
C1—H1	0.9300	C11—H11A	0.9600
C2—H2	0.9300	C11—H11B	0.9600
C3—C4	1.392 (3)	C11—H11C	0.9600
C3—H3	0.9300	C12—H12A	0.9600
C4—C5	1.415 (3)	C12—H12B	0.9600
C4—H4	0.9300	C12—H12C	0.9600
C5—C6	1.475 (3)		
			
C2—N1—C3	121.81 (19)	C10—C7—C8	112.8 (2)
C2—N1—H1D	119.1	C12—C8—N3	110.0 (2)
C3—N1—H1D	119.1	C12—C8—C7	117.4 (2)
O1—N2—C6	126.78 (18)	N3—C8—C7	100.76 (18)
O1—N2—C7	120.83 (19)	C12—C8—C11	109.7 (3)
C6—N2—C7	112.09 (17)	N3—C8—C11	105.4 (2)
O2—N3—C6	126.73 (17)	C7—C8—C11	112.6 (2)
O2—N3—C8	120.85 (18)	C7—C9—H9A	109.5
C6—N3—C8	112.09 (17)	C7—C9—H9B	109.5
C2—C1—C5	119.6 (2)	H9A—C9—H9B	109.5
C2—C1—H1	120.2	C7—C9—H9C	109.5
C5—C1—H1	120.2	H9A—C9—H9C	109.5
N1—C2—C1	120.7 (2)	H9B—C9—H9C	109.5
N1—C2—H2	119.6	C7—C10—H10A	109.5
C1—C2—H2	119.6	C7—C10—H10B	109.5
N1—C3—C4	120.6 (2)	H10A—C10—H10B	109.5
N1—C3—H3	119.7	C7—C10—H10C	109.5
C4—C3—H3	119.7	H10A—C10—H10C	109.5
C3—C4—C5	119.5 (2)	H10B—C10—H10C	109.5
C3—C4—H4	120.2	C8—C11—H11A	109.5
C5—C4—H4	120.2	C8—C11—H11B	109.5
C4—C5—C1	117.69 (18)	H11A—C11—H11B	109.5
C4—C5—C6	121.17 (19)	C8—C11—H11C	109.5
C1—C5—C6	121.13 (19)	H11A—C11—H11C	109.5
N3—C6—N2	108.00 (17)	H11B—C11—H11C	109.5
N3—C6—C5	125.80 (18)	C8—C12—H12A	109.5
N2—C6—C5	126.18 (19)	C8—C12—H12B	109.5
N2—C7—C9	110.2 (2)	H12A—C12—H12B	109.5
N2—C7—C10	105.5 (2)	C8—C12—H12C	109.5
C9—C7—C10	109.9 (2)	H12A—C12—H12C	109.5
N2—C7—C8	101.19 (18)	H12B—C12—H12C	109.5
C9—C7—C8	116.3 (2)		
			
C3—N1—C2—C1	1.1 (3)	C6—N2—C7—C9	143.3 (2)
C5—C1—C2—N1	1.0 (3)	O1—N2—C7—C10	76.0 (3)
C2—N1—C3—C4	−1.1 (3)	C6—N2—C7—C10	−98.1 (2)
N1—C3—C4—C5	−0.9 (3)	O1—N2—C7—C8	−166.4 (2)
C3—C4—C5—C1	2.9 (3)	C6—N2—C7—C8	19.6 (2)
C3—C4—C5—C6	−176.24 (19)	O2—N3—C8—C12	−40.2 (3)
C2—C1—C5—C4	−2.9 (3)	C6—N3—C8—C12	146.0 (2)
C2—C1—C5—C6	176.2 (2)	O2—N3—C8—C7	−164.7 (2)
O2—N3—C6—N2	176.6 (2)	C6—N3—C8—C7	21.4 (2)
C8—N3—C6—N2	−10.0 (2)	O2—N3—C8—C11	78.0 (3)
O2—N3—C6—C5	−4.9 (3)	C6—N3—C8—C11	−95.8 (2)
C8—N3—C6—C5	168.50 (19)	N2—C7—C8—C12	−141.9 (2)
O1—N2—C6—N3	179.6 (2)	C9—C7—C8—C12	98.7 (3)
C7—N2—C6—N3	−6.8 (2)	C10—C7—C8—C12	−29.7 (3)
O1—N2—C6—C5	1.1 (4)	N2—C7—C8—N3	−22.6 (2)
C7—N2—C6—C5	174.72 (19)	C9—C7—C8—N3	−142.0 (2)
C4—C5—C6—N3	8.4 (3)	C10—C7—C8—N3	89.7 (2)
C1—C5—C6—N3	−170.7 (2)	N2—C7—C8—C11	89.3 (2)
C4—C5—C6—N2	−173.4 (2)	C9—C7—C8—C11	−30.1 (3)
C1—C5—C6—N2	7.5 (3)	C10—C7—C8—C11	−158.4 (2)
O1—N2—C7—C9	−42.7 (3)		

### Hydrogen-bond geometry (Å, °)

**Table:**

*D*—H···*A*	*D*—H	H···*A*	*D*···*A*	*D*—H···*A*
N1—H1D···Cl1^i^	0.86	2.17	3.028 (3)	174

Symmetry codes: (i) *x*+1, *y*+1, *z*.
